# Liquid Metal-Based Dual-Response Pressure Sensor for Dual-Modality Sensing and Robotic Object Recognition

**DOI:** 10.3390/bioengineering11121211

**Published:** 2024-11-29

**Authors:** Yanru Bai, Zhi Wang, Yizhuo Zhang, Rui Guo, Xisheng Li

**Affiliations:** 1 School of Automation and Electrical Engineering, University of Science and Technology Beijing, Beijing 100083, China; yrbai@sina.com; 2 School of Advanced Engineering, University of Science and Technology Beijing, Beijing 100083, China; 3 School of Precision Instrument and Opto-Electronics Engineering, Tianjin University, Tianjin 300072, China; dpgd1006@163.com (Z.W.); zoezyz002@gmail.com (Y.Z.)

**Keywords:** liquid metal, pressure sensor, object recognition

## Abstract

Characterized by their high sensitivity and flexible deformation, flexible pressure sensors have been extensively applied in various fields such as wearable electronics, health monitoring, soft robotics, and human–computer interaction. In this research, we developed a dual-response pressure sensor (DRPS) designed to identify object materials. By integrating the operating principles of capacitive and resistive sensors and employing microstructured dielectric layers, we enhanced the sensitivity and detection range of the pressure sensor. Additionally, this research introduced an innovative, simple, and cost-effective method for preparing flexible pressure sensors. Following a comprehensive performance evaluation, the DRPS exhibited high sensitivity, a broad detection range, and robust stability. Finally, utilizing a mechanical claw equipped with an intelligent perception data collection system, we effectively distinguished various materials, further corroborating the practicality of DRPS in intelligent perception applications.

## 1. Introduction

Flexible pressure sensors have garnered unprecedented attention and have been extensively applied in various fields such as the Internet of Things, wearable electronics, intelligent robotics, and human–computer interaction. These sensors reflect the magnitude and distribution of external pressures by converting pressure into electrical signals through mechanisms like piezoelectric effects or resistance changes. Due to their excellent pressure-sensing capabilities and flexibility, flexible pressure sensors have become a primary focus in interdisciplinary research involving materials science, electronic engineering, and biomedicine [1]. For instance, when robot hands covered with flexible pressure sensor arrays grasp an object, these sensors accurately detect the pressure at each contact point because of their high sensitivity [2]. Based on these pressure data, the robot’s control system can adjust its grip strength in real time, achieving a human-like grasp. Therefore, pressure sensors must possess high precision (with errors less than 1%), reliability, and millisecond-level response times [3]. However, traditional rigid pressure sensors lack the high sensitivity, spatial resolution, and rapid response characteristic of human skin perception [4]. Moreover, being made from materials like silicon and metal films, their rigidity makes them ill suited for non-planar or dynamically changing surfaces. In contrast, flexible pressure sensors often use organic materials and thin films with good elasticity and bendability [5]. These materials not only conform to irregular surfaces but also maintain high sensitivity and stability while enabling more precise data collection and monitoring. Thus, flexible pressure sensors demonstrate irreplaceable value in intelligent sensing domains such as advanced robotics, wearable devices, and biomedical engineering.

Currently, flexible pressure sensor detection principles mainly include piezoresistive, capacitive, piezoelectric, and triboelectric responses. Piezoresistive sensors detect pressure through changes in material resistance [6,7,8,9]; capacitive sensors sense pressure via capacitance variations [10,11,12,13]; piezoelectric sensors monitor pressure using voltage changes generated by the material [14,15,16,17,18,19]; and triboelectric sensors track pressure changes based on the triboelectric effect [20,21,22,23]. Among them, resistive flexible pressure sensors are advantageous due to their simple device structure, easy manufacturing process, and wide detection range [24,25]. Capacitive flexible pressure sensors boast high sensitivity, low power consumption, and minimal dependence on temperature and humidity variations [26]. However, flexible pressure sensors that rely on a single detection principle struggle to achieve high sensitivity and a broad pressure detection range simultaneously. Although integrated sensing elements or sensor arrays offer improved performance, they require complex structures and challenging manufacturing processes [27,28,29,30,31]. Consequently, the high cost of materials, complex manufacturing techniques, and stringent precision requirements make these flexible pressure sensors difficult to implement in practical applications. Hence, reducing production costs and simplifying fabrication processes while maintaining good performance are the key focuses in current flexible sensor research.

Unlike common rigid metal materials, liquid metals like gallium–indium alloy possess excellent plasticity, making them stretchable and deformable [32]. Although mercury is a well-known liquid metal, its toxicity has led to its avoidance in practical applications. Gallium–indium alloy is gaining popularity due to its lower toxicity and melting point below room temperature. Their good conductivity [33], thermal conductivity [34], and biocompatibility [35] make them an ideal material for a flexible pressure sensor, finding widespread use in wearable sensors [36], batteries [37], flexible robots [38], and human–machine interaction [39].

To address the limited sensitivity and detection range of existing flexible pressure sensors, this study innovatively combined capacitive and resistive sensing technologies to develop a novel dual-response pressure sensor (DRPS). This sensor achieves both high sensitivity and a wide detection range, making it suitable for various intelligent pressure-sensing scenarios. Compared with previous work, the sensor has the same order of detection sensitivity, detection range, and response time [40,41,42]. Unlike traditional methods like lithography and sputtering, the fabrication method used in this study is cost-effective and straightforward. Additionally, its fabrication utilizes various flexible materials, allowing the sensor to adapt to mechanical claw surfaces and reflect applied pressure in real time while maintaining high stability after multiple uses. Furthermore, we developed a mechanical claw pressure-sensing system integrated with DRPS and employed machine learning algorithms to analyze the collected pressure data in detail. This enables the mechanical claw to accurately recognize objects made of different materials.

## 2. Materials and Methods

### 2.1. Preparation of Cu-EGaIn

Firstly, liquid metal was made by mixing gallium and indium metals (Anhui Minor New Materials Co., Ltd., Chuzhou, China) with mass fractions of 75.5% and 24.5%, respectively. Then, 15 g of copper microparticles (average diameter of 15 μm, Hebei Jingrui Alloy Products Co., Ltd., Hebei, China) was added to a beaker containing 100 g of liquid metal, followed by the addition of 1 mol/L of NaOH solution. With continuous stirring using glass rods, the copper microparticles gradually integrated into the liquid metal to obtain Cu-EGaIn.

### 2.2. Preparation of Resistance Pressure Sensor

An Ecoflex mixture (Ecoflex 00-30, Smooth-On Inc., Macungie, PA, USA) was made by mixing two prepolymers in a mass ratio of 1:1. Then, the mixture was poured onto a glass plate and spun into a film with a thickness of 100 μm using a spin coater (KW-4A-120S, Shanghai Kemet functional ceramic Technology Co., Ltd., Shanghai, China). Next, the Ecoflex film was cured by heating at 60 °C for 10 min. A stainless steel template was placed over the film, and then Cu-EGaIn was roller-coated onto the stainless steel template. After removing the template, the Cu-EGaIn wire was adhered to the Ecoflex film. Two copper tapes were applied to both ends of the wire for connection with other circuits. Finally, the Ecoflex mixture was poured onto the Cu-EGaIn wire, and cured by heating at 60 °C for 10 min to complete the packaging of the resistance pressure sensor.

### 2.3. Preparation of Capacitive Pressure Sensor

The capacitive pressure sensor was assembled using a typical “sandwich” structure. The bottom electrode (unit area: 5 × 5 mm^2^) was printed on an Ecoflex film and coated with a layer of uncured film using the same preparation method for the resistance pressure sensor. At the same time, PDMS was poured into a 3D-printed anti-micropyramid array template, and the bubbles were removed in a vacuum-drying oven to fill the solution. The PDMS was cured by heating at 100 °C for 2 h, and it was easy to peel off and obtain a PDMS film with a micropyramid array (6 × 6 matrix). Then, this array was placed on the bottom electrode covered with an uncured Ecoflex film, and the two parts were combined by heating at 60 °C for 10 min. Similarly, the top electrode was printed on an Ecoflex film using the same preparation method for the resistance pressure sensor, and the bottom surface of the film was coated with a layer of uncured film. The other side of the micropyramid array was placed on the Ecoflex film printed with the top electrode, and the two parts were combined by heating at 60 °C for 10 min. After that, the upper surface of the top electrode of the capacitive pressure sensor was coated with a layer of uncured Ecoflex film. Finally, the capacitive and resistance pressure sensors were bonded by heating at 60 °C for 10 min. The pressure sensor was fixed to the glove by applying adhesive glue (MS-21-1, Mingsheng Rubber Co., Ltd., Shanghai, China) at their interface.

### 2.4. Characterization and Measurements

A tensile tester (HC-01, Dongtai suheng Transmission Technology Co., Ltd., Danyang, China) was used to measure the mechanical properties of the sensor. The capacitance measurements were recorded on an Agilent (E4980A, Agilent Technologies Co.Ltd., Shanghai, China) Precision LCR meter. The resistance test method was used to measure the resistance change in the sensor using a digital source meter (Keithley 2002, Tektronix, Inc., Beaverton, OR, USA). Scanning electron microscopy (SEM, Apreo, Thermo Fisher Technology Co., Ltd., Shanghai, China) was used to characterize the microstructure of Cu-EGaIn.

## 3. Results and Discussion

### 3.1. Design and Fabrication of DRPS

**Figure 1:**
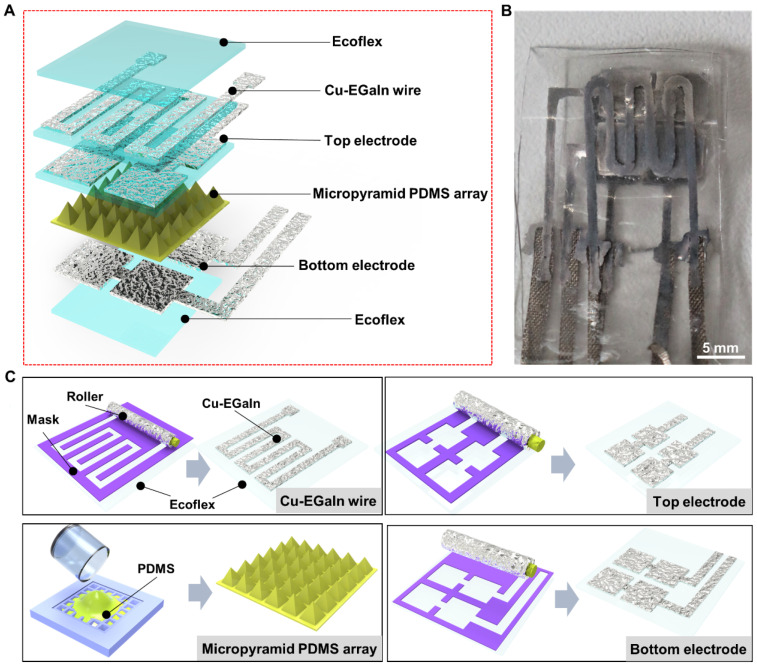
Design and fabrication of DRPS. (**A**) Schematic diagram of the DRPS consisting of a capacitive pressure sensor and a resistive pressure sensor. (**B**) Optical image of the DRPS. (**C**) The fabrication of DRPS.

This DRPS consists of a resistive pressure sensor and a capacitive pressure sensor, as illustrated in Figure 1A. The resistive pressure sensor is made up of a microchannel filled with liquid metal that deforms under pressure, resulting in a change in the resistance of the internal liquid metal. The capacitive pressure sensor employs a traditional sandwich structure, utilizing a microstructured dielectric layer to enhance its sensitivity. Additionally, the upper and lower plates of the capacitive pressure sensor are composed of individual electrodes, providing four-channel capacitance signals to further improve the accuracy of pressure detection. Here, both the upper and lower plates are prepared by coating liquid metal onto Ecoflex films using a template printing method (Figure S1). Research has shown that liquid metal, due to its high surface tension, is difficult to coat uniformly on Ecoflex film surfaces, and the coating formed tends to be thick [43]. To address this issue, this study used silver-coated copper particles as dopants, mixed into gallium-based liquid metal at a weight ratio of 15% to create a semi-liquid metal material (Cu-EGaIn), as shown in Figure S2. The Cu-EGaIn exhibits better printability than pure liquid metal, and the incorporated copper particles significantly reduce its fluidity, preventing noticeable deformation under gravity. From the SEM characterization (Figure S3), it can be observed that numerous copper particles are dispersed within and encapsulated by the liquid metal. The oxide layer formed on the surface of the liquid metal exposed to air also restricts its flow. Figure 1B displays photographs of the DRPS components. Considering the material requirements of the integrated sensor, along with the goals of flexibility, miniaturization, and minimizing fabrication difficulty and cost, the sensor’s design specifications are as follows: each electrode measures 10 mm × 10 mm × 150 μm, the flexible dielectric is a 6 × 6 micropyramid array sensor with dimensions of 2.5 mm × 2.5 mm × 600 μm, and the overall size is 12 mm × 12 mm × 2 mm. The thickness of the intermediate dielectric layer not only gives the upper plate a larger deformation space, but also provides a suitable PDMS response and recovery time. Figure 1C illustrates the fabrication process of the DRPS, which includes the following three steps: printing the liquid metal conductors for the resistive pressure sensor, manufacturing the micropyramid array, and printing the liquid metal plates for the capacitive pressure sensor. Details of the fabrication process are further described in the Section 2. Since all components of the DRPS are made from soft materials, it can undergo significant deformation under pressure, thereby achieving substantial variations in the distance between capacitive plates and the shape of the resistive sensor.

### 3.2. Characterization of Capacitive Pressure Sensor

**Figure 2:**
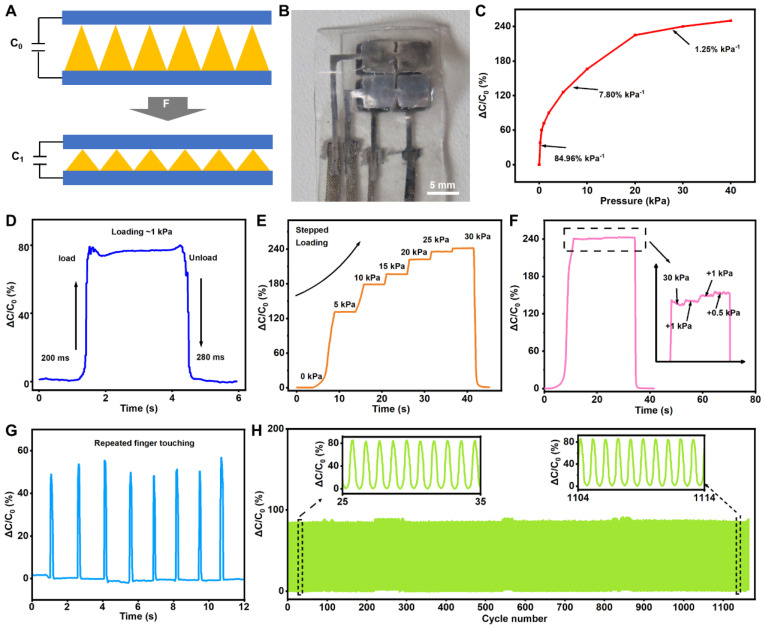
Characterization of capacitive pressure sensors. (**A**) Schematic diagram of the capacitive pressure sensor structure under applied pressure. (**B**) Optical images of the capacitive pressure sensor. (**C**) Capacitance variation in the capacitive pressure sensor at different pressures. (**D**) Instant response time of the capacitive pressure sensor. (**E**) Capacitance response to stepped loading of the capacitive pressure sensor. (**F**) Detection of micro-pressure under loading pressures of 30 kPa. (**G**) Transient response of the capacitive pressure sensor to the repeated finger-touching process. (**H**) Stability of the capacitive pressure sensor under repeated pressure (1 kPa) over 1114 cycles.

The capacitance variation in the capacitive pressure sensor arises from changes in the distance between its upper and lower plates and the dielectric constant of the dielectric layer, as illustrated in Figure 2A. The integration of a micropyramid array with a flexible liquid metal plate facilitates the capacitive pressure sensor’s high sensitivity, as depicted in Figure 2B. Here, the sensitivity is defined as *S* = (Δ*C*/*C*)/Δ*P*, where *S* is the sensor’s sensitivity of the sensor, *C* is its capacitance, and *P* is the applied pressure. *C* can be calculated using the following formula: *C* = *ϵA*/*d*, where *ϵ* is the dielectric constant, *A* is the area of the electrode, and *d* is the distance between the two electrodes.

This type of capacitive pressure sensor has a detection range of up to 40 kPa, exhibiting significant variations in sensitivity across different pressure ranges, as shown in Figure 2C. A maximum sensitivity of 84.96% kPa^−1^ is observed at pressures below 1 kPa, while a reduced response corresponds to a sensitivity of up to 7.8% kPa^−1^ over a pressure range of 1–20 kPa, and a broad pressure detection range of 150 kPa is noted. As the applied pressure progressively increases, the sensor’s sensitivity diminishes. This phenomenon occurs due to the reduced deformation of the intermediate medium layer under higher pressure conditions, leading to a comparative decrease in the magnitude of capacitance change and, consequently, a decline in sensitivity. Therefore, this capacitive flexible pressure sensor is suitable for measuring minute pressures and possesses high sensitivity under lower pressures. Response time, defined as the duration required for the flexible pressure sensor’s signal to reach 90% of its stable output, and recovery time, the interval needed for the sensor’s signal to return to its initial value after the applied pressure is removed, are crucial parameters for assessing the sensor’s real-time dynamic pressure- sensing performance. In the realm of intelligent sensing, a shorter response time is preferable. This study employed weights to measure the response time by placing a weight of known mass on the sensor during testing. Due to the good resilience of PDMS, which can quickly recover its initial state after pressure removal, the capacitive pressure sensor demonstrates a rapid response of less than 280 milliseconds under a pressure of 1 kPa. This finding indicates its robust dynamic responsiveness and ability to swiftly detect pressure changes (Figure 2D). Because of the microstructure’s capability to store and release energy for elastic recovery, the pressure sensor can accurately measure pressure under continuous variation (from 0 to 30 kPa, in steps of 5 kPa), as shown in Figure 2E. As pressure increases linearly, the sensor’s output capacitance gradually rises, maintaining a consistent value under the same pressure. Moreover, Figure 2F shows that the sensor can detect slight pressure changes when additional pressures of 1 and 0.5 kPa are progressively added at 30 kPa, indicating that it can still recognize minor fluctuations at higher pressures (30 kPa). These results demonstrate the sensor’s capability to discern small pressures amidst larger ones. Additionally, the sensor’s upper surface was repeatedly pressed using a human finger. The outcomes presented in Figure 2G indicate that the capacitive pressure sensor can maintain stable capacitance change signals. To verify the sensor’s dynamic stability, it was subjected to approximately 1150 repeated loads with a force of ≈1 kPa (Figure 2H). The sensor exhibits an exceptionally stable signal output, underscoring its outstanding stability and suitability for prolonged usage. These superior characteristics suggest that the sensor can achieve high-sensitivity pressure detection, making it ideal for applications such as robotic tactile sensing.

### 3.3. Characterization of Resistive Pressure Sensors

**Figure 3:**
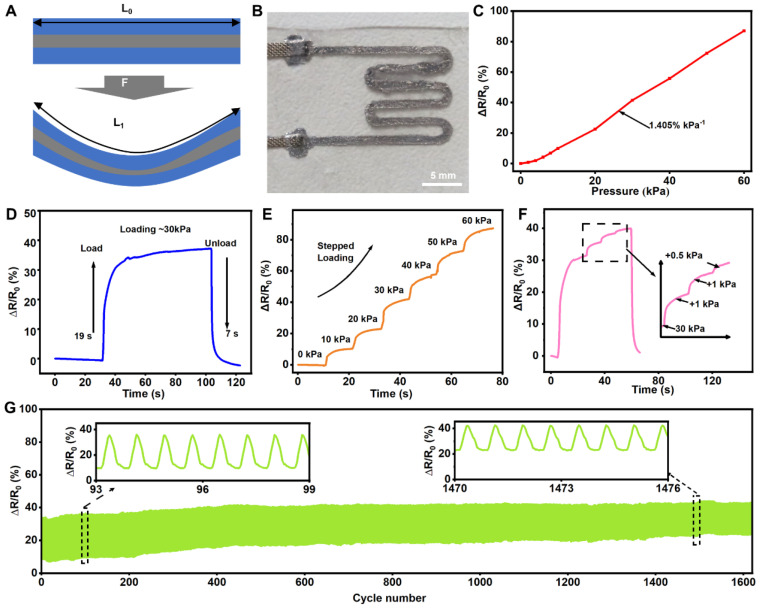
Characterization of resistive pressure sensor. (**A**) Schematic diagram of the resistive pressure sensor structure under applied pressure. (**B**) Optical images of the resistive pressure sensor. (**C**) Resistive variation in the resistive pressure sensor at different pressures. (**D**) Response time of the resistive pressure sensor. (**E**) Resistive response to stepped loading of the resistive pressure sensor. (**F**) Detection of micro-pressure under loading pressures of 30 kPa. (**G**) Stability of the resistive pressure sensor under repeated pressure (20 kPa) over 1600 cycles.

The resistance variation in the resistive pressure sensor originates from the shape changes in the conductive path, as illustrated in Figure 3A. Under external pressure, the cross-sectional area of the liquid metal wire decreases while its length increases, leading to an increase in its resistance value (Figure 3B). Here, the sensitivity *S* of the resistive pressure sensor is defined as *S* = *δ*(Δ*R*/*R*0)/*δP*, where Δ*R* = *R* − *R*0 represents the response resistance of the sensor under pressure, *R*0 is the initial resistance, and *P* is the applied pressure. As shown in Figure 3C, the change in resistance of the resistive flexible pressure sensor exhibits a linear relationship with applied pressure. Within the pressure range of 0 kPa to 60 kPa, the sensor’s sensitivity remains constant at approximately 1.405% per kPa. Thus, this resistive flexible pressure sensor features good linearity and a wide measurement range, making it suitable for measuring pressure under high-pressure conditions.

After placing a 300 g weight, the resistive flexible pressure sensor stabilizes at a constant value after 19 s and quickly returns to its initial resistance value within 7 s after the weight is removed (Figure 3D). Compared with the response time of the capacitive pressure sensor, that of the resistive pressure sensor is too slow for rapid reactions to external pressures. Therefore, in practical applications, capacitive pressure sensors are needed to complement resistive pressure sensors in order to enhance the response speed to high pressures. The pressure applied to the resistive pressure sensor is gradually increased from 0 kPa to 60 kPa, with a pause at each 10 kPa increment. An analysis of experimental data reveals that the resistance change progressively increases with increasing applied pressure, maintaining consistency at each 5 kPa increment. This result clearly demonstrates the good linearity of the resistive pressure sensor over a wide measurement range, as shown in Figure 3E. To test whether the resistive pressure sensor can provide sufficient sensitivity and resolution under high-pressure conditions, it was subjected to a pressure of 30 kPa, followed by incremental loads of 1 kPa and 0.5 kPa. Figure 3F indicates that the flexible pressure sensor can still detect minor pressure changes under high pressure and respond with signal variations. The results demonstrate the sensor’s ability to recognize small pressure changes when subjected to high pressures. Finally, to verify the cyclic stability of the resistive pressure sensor, we applied a force of 20 kPa to the sensor for 1600 repeated cycles. Figure 3G shows that the output signal of the resistive pressure sensor experiences only a slight drift.

### 3.4. Applications of DRPS

**Figure 4:**
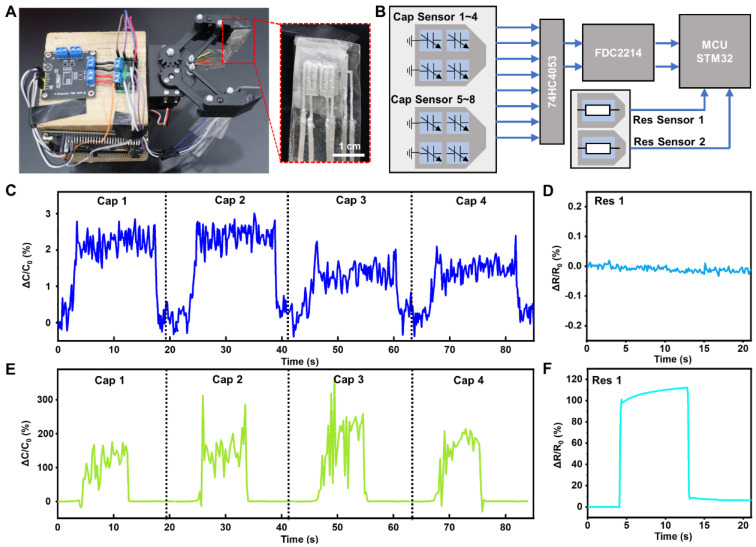
Pressure detection range of DRPS for mechanical claw pressure sensing. (**A**) Optical images of the DRPS attached on a mechanical claw. (**B**) Single amplifier reading circuits. (**C**) Capacitance response of four capacitive pressure sensors under loading pressure of 8 Pa. (**D**) Resistive response of the resistive pressure sensors under loading pressure of 8 Pa. (**E**) Capacitance response of four capacitive pressure sensors under loading pressure of 100 kPa. (**F**) Resistive response of the resistive pressure sensors under loading pressure of 100 kPa.

In this study, we affixed a sensor to the robotic hand, enabling it to perceive pressure, as illustrated in Figure 4A. The initial capacitance and capacitance variations in the flexible capacitive sensor are at the pF level, demanding high precision in testing. Consequently, we employed a capacitance detection chip (FDC22214, Xiamen Xinwang Electronic Technology Co., Ltd., Xiamen, China) to measure the DRPS capacitance values. The initial resistance of the resistive flexible pressure sensor is approximately several ohms; hence, we utilized a resistance-measuring instrument (CXT2518, Xiamen Xinwang Electronic Technology Co., Ltd., Xiamen, China) to monitor changes in resistance values in real time. An analog switch (74HC4053, Xiamen Xinwang Electronic Technology Co., Ltd., Xiamen, China) was used to select individual capacitive sensors, after which the selected capacitor’s two plates were connected to the detection terminals of the capacitance detection chip; the results were output to a microcontroller (STM32, STMicroelectronics N.V., Geneva, Switzerland) and fed into a computer, as shown in Figure 4B. During capacitance measurement, since the values are at the pF level, they are susceptible to stray capacitance, which varies with structure and position and complicates measurements. Therefore, each measurement first subtracts the stray and reference capacitance values, a dynamic compensation method that effectively eliminates the impact of stray capacitance. This measurement circuit requires 40 ms to capture one capacitance value, allowing for the acquisition of eight capacitance values on the mechanical claw every 160 ms. To explore the detection threshold of the flexible pressure sensor, and its ability to perceive minute pressures, we placed an ultra-light paper weighing 0.08 g on the sensor, exerting a pressure of approximately 8 Pa. Figure 4C,D show that placing the ultra-light paper on the capacitive pressure sensor causes a noticeable change in the capacitance value (about 2.5%), which rapidly returns to its initial state upon removal of the paper. In contrast, the output of the resistive pressure sensor remains virtually unchanged. This indicates that capacitive flexible pressure sensors are highly sensitive to minute pressures and respond quickly. To investigate the upper detection limit of flexible pressure sensors, a 1 kg weight was placed on the device, exerting a pressure of about 100 kPa. From Figure 4E, it is evident that after placing the weight on the sensor, its capacitance value changes significantly but is extremely unstable. This instability is attributed to severe deformation of the capacitive pressure sensor’s intermediate dielectric layer under immense pressure, which compromises the sensor’s basic structure and renders its capacitance output unable to accurately reflect the applied pressure. The liquid metal electrode also has obvious deformation under high pressure, and the difference in electrode shape and conductivity affects the stability of the capacitor signal. Conversely, the resistive pressure sensor’s output signal remains stable under high pressure, accurately representing the applied pressure (Figure 4F). Thus, combining capacitive and resistive flexible pressure sensors leverages the advantages of both sensing mechanisms, creating a flexible pressure sensor with a broad range, high sensitivity, and excellent stability.

**Figure 5:**
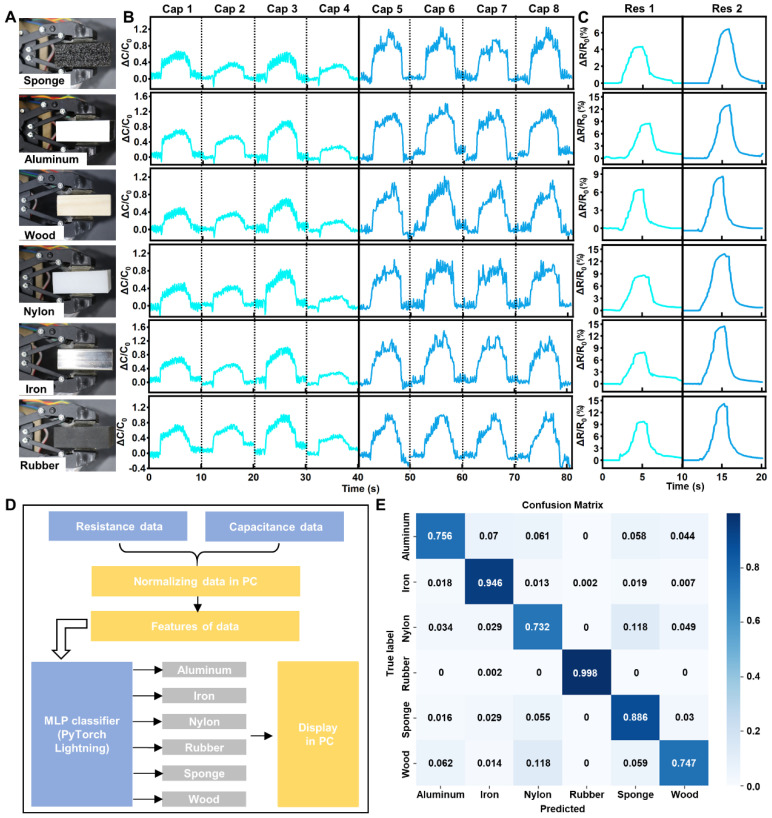
The DRPS for object recognition in robotic hands. (**A**) Optical images of the robotic hand with six kinds of materials. (**B**) Eight capacitance and two resistance (**C**) data for six kinds of materials. (**D**) The flow diagram of machine learning algorithm. (**E**) The confusion matrix of evaluation results.

Finally, we demonstrate the application of this pressure sensor in enabling a robotic hand to recognize the material of grasped objects. For this purpose, we tested six different materials (sponge, aluminum, wood, nylon, rubber, and an iron block) of identical shapes (40 mm × 40 mm × 20 mm). This effectively eliminated the influence of object shape on material perception accuracy, ensuring the sensor’s response primarily correlated with the material type, as shown in Figure 5A. Each material was grasped 20 times, collecting 8 capacitance (Figure 5B) and 2 resistance (Figure 5C) data for the 6 material types. Subsequently, we employed machine learning algorithms (Multi-layer Perceptron, MLP) to process and analyze the collected capacitance and resistance data for object recognition, as shown in Figure 5D. The evaluation results are presented in the confusion matrix displayed in Figure 5E. The prediction precision results for the six materials are 88.6%, 75.6%, 74.7%, 73.2%, 99.8%, and 94.6%, respectively.

## 4. Conclusions

In this study, we combined two types of sensors to leverage their respective advantages. The capacitive flexible pressure sensor is utilized for precisely detecting micro-pressures, while the resistive flexible pressure sensor is suitable for measuring a broader range. By integrating both sensors, we can achieve a sensor with high sensitivity and an extensive detection range. Furthermore, due to the differing operational mechanisms of these two sensors, their respective sources of systematic errors also exhibit variation. Utilizing cross-comparison and the fusing data obtained from both sensors, we can mutually validate measurement outcomes and thereby augment precision and dependability. In subsequent research, we will explore other structures to improve the sensitivity of the pressure sensors, such as introducing microstructures on flexible substrates or using composite materials as conductive layers. The size of current sensors can be further optimized and affixed to each finger of the robotic hand, enabling multidimensional pressure perception to recognize a wider variety of materials and shapes. In addition, the sensors can be affixed to human skin to achieve limb joint collision detection, especially under high pressure, or to the sole and surface of sports shoes to achieve gait detection and other functions.

## Data Availability

The original contributions presented in this study are included in the article/Supplementary Materials. Further inquiries can be directed to the corresponding authors.

## Supplementary Materials

The following supporting information can be downloaded at: https://www.mdpi.com/article/10.3390/bioengineering11121211/s1, Figure S1: Optical images of the preparation of DRPS; Figure S2: Optical images of the preparation of Cu-EGaIn; Figure S3: SEM image of Cu-EGaIn.
