# Supplementary material for: Liquid Metal-Based Dual-Response Pressure Sensor for Dual-Modality Sensing and Robotic Object Recognition
Source: Bioengineering (Basel). 2024 Nov 29;11(12):1211. doi: 10.3390/bioengineering11121211 (PMC11673121; doi:10.3390/bioengineering11121211)
Supplement: Supplementary file 1 [file bioengineering-11-01211-s001.zip › bioengineering-3321109-supplementary.pdf]

Liquid Metal-Based Dual-Response Pressure Sensor for Dual-Modality Sensing and Robotic Object Recognition

Yanru Bai [1,2,†], Zhi Wang [3,†], Yizhuo Zhang [3], Rui Guo [3,*] and Xisheng Li [1,*]

1 School of Automation and Electrical Engineering, University of Science and Technology Beijing, Beijing 100083, China; yrbai@sina.com
2 School of Advanced Engineering, University of Science and Technology Beijing, Beijing 100083, China
3 School of Precision Instrument and Opto-Electronics Engineering, Tianjin University, Tianjin 300072, China; dpgd1006@163.com (Z.W.); zoezyz002@gmail.com (Y.Z.)
* Correspondence: guorui21@tju.edu.cn (R.G.); lxs@ustb.edu.cn (X.L.)
† These authors contributed equally to this work.

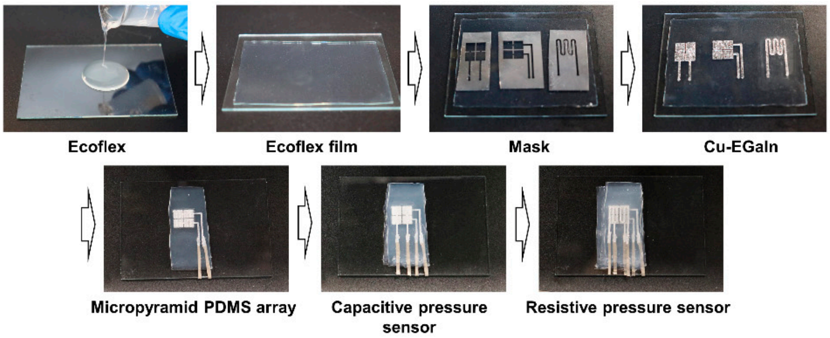

Figure S1. Optical images of the preparation of DRPS.

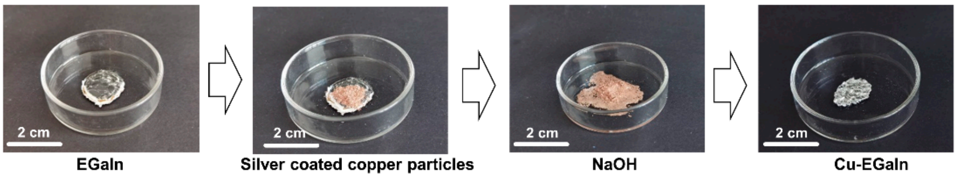

Figure S2. Optical images of the preparation of Cu-EGaIn.

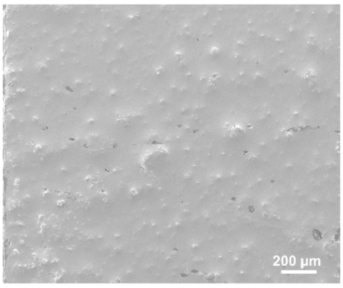

Figure S3. SEM image of Cu-EGaIn.
